# Improvement of both fasting and postprandial glycemic control by the two-step addition of miglitol and mitiglinide to basal insulin therapy: a pilot study

**DOI:** 10.1186/1758-5996-6-48

**Published:** 2014-03-31

**Authors:** Noriko Ihana, Tetsuro Tsujimoto, Ritsuko Yamamoto-Honda, Miyako Kishimoto, Hiroshi Kajio, Hiroshi Noto, Masafumi Kakei, Mitsuhiko Noda

**Affiliations:** 1 Department of Diabetes, Endocrinology and Metabolism, National Center for Global Health and Medicine Center Hospital, Tokyo, Japan; 2 Division of General Medicine, Jichi Medical University Graduate School of Medicine, Tochigi, Japan; 3 First Department of Comprehensive Medicine, Saitama Medical Center, Jichi Medical University School of Medicine, Saitama, Japan; 4 Department of Diabetes Research, Diabetes Research Center, National, Center for Global Health and Medicine, Tokyo, Japan

**Keywords:** Miglitol, Mitiglinide, Insulin glargine, Postprandial hyperglycemia, Glucose fluctuation, Continuous glucose monitoring (CGM)

## Abstract

**Background:**

Combination therapy consisting of basal insulin and oral hypoglycemic agents (OHAs) is effective for the treatment of type 2 diabetes (T2DM) that cannot be adequately controlled using OHAs alone. Though basal insulin with metformin or sulfonylurea is an effective therapy, it cannot reduce postprandial glycemia without the risk of hypoglycemia. We examined a two-step regimen consisting of the addition of postprandial hypoglycemic agents (an alpha-glucosidase inhibitor and a glinide) in patients whose T2DM was poorly controlled using basal insulin therapy.

**Methods:**

Inpatients between the ages of 30–79 years who had T2DM and an HbA1c level of more than 7.0% were recruited. The patients were treated with once-daily insulin glargine with or without metformin, depending on the patient’s age and renal function. Insulin glargine was titrated to achieve a target fasting glucose level of 70–130 mg/dL as a first step (STEP0). If the 2-hour postprandial glucose (PBG) level was higher than the target of 180 mg/dL, miglitol treatment (150 mg/day) was initiated, with dose adjustments (75–225 mg) allowed depending on abdominal symptoms and the PBG (STEP1). If the PBG of the patients remained higher than the target after 3 days of treatment, mitiglinide (30 mg/day, titrated up to 60 mg) was added (STEP2). We then evaluated the proportion of patients who achieved the target PBG before and after the two-step regimen. Continuous Glucose Monitoring (CGM) was performed throughout the two-step protocol in most of the patients.

**Results:**

Of the 16 patients who were recruited (median age, 67.0 [58.0-71.0] years; body mass index, 25.0 [22.0-27.9] kg/m^2^; HbA1c level at admission, 9.1% [8.35-10.4%]), 1 patient (6.25%) achieved the target PBG at STEP 0 and 14 patients (87.5%) had achieved the target PBG at the end of the treatment protocol (*P* = 0.002). CGM showed a significant decrease in the glucose level at each step of the protocol. The standard deviations in the CGM glucose levels for 24 hours, MAGE, and M-value also improved.

**Conclusions:**

The two-step addition of postprandial hypoglycemic agents to basal insulin therapy is potentially effective and safe for decreasing both the fasting and postprandial glucose levels.

## Background

Type 2 diabetes is characterized by both decreased insulin secretion and sensitivity [1]. The impairment of insulin secretion derived from β cell dysfunction is a progressive process that occurs before diagnosis and throughout the course of type 2 diabetes [2,3]. Therefore, many patients who are initially treated with oral hypoglycemic agents (OHAs) eventually require a treatment strategy that includes insulin to counteract persistent hyperglycemia.

One treatment strategy that includes insulin is the combination of basal insulin with oral hypoglycemic agents. Historically, a regimen combining basal insulin with sulfonylurea and metformin has been frequently used. Using this regimen, the insulin dose can be easily titrated to minimize the number of hypoglycemic episodes and weight gain, compared with multiple insulin injections or mixed insulin therapy [4,5]. However, a drawback of this regimen is that it provides poor support against sustained post-breakfast hyperglycemia (a situation that is often observed in patients with poor glycemic control [6,7]) and postprandial hyperglycemia, resulting in glucose fluctuations that can increase the risks of both cardiovascular disease [8-11] and microvascular disease [12,13]. Attempts to lower the postprandial glucose level by increasing the dose of basal insulin can lead to an increased risk of severe hypoglycemia during the night and early morning, which might also be associated with an increased risk of cardiovascular disease [14,15].

Accordingly, we have proposed a regimen consisting of a two-step administration of postprandial hypoglycemic agents, namely an alpha-glucosidase inhibitor and a glinide, to basal insulin therapy to target postprandial hyperglycemia. We then conducted a pilot study to examine the safety and efficacy of this regimen.

## Methods

### Patients

We enrolled patients with type 2 diabetes who were hospitalized at the National Center for Global Health and Medicine Center Hospital between November 2011 and August 2013. Candidate subjects were between the ages of 30–79 years and had insufficient glucose control (HbA1c ≧ 7.0%). Patients with severe renal dysfunction (estimated glomerular filtration rate <30 mL/min/1.73 m^2^ or continuous hemodialysis), severe liver dysfunction, type 1 diabetes, glutamic acid decarboxylase antibody positivity, malignancy, or other causes of hyperglycemia were excluded from the present study.

All the patients provided written informed consent a few days after hospitalization and prior to enrollment in the trial. The study protocol was approved by the ethics committee of the National Center of Global Health and Medicine Center Hospital and was implemented in accordance with the provisions of the Declaration of Helsinki.

### Study design

#### ***STEP 0***

First, patients were treated with diet therapy (25–30 kcal/ideal body weight/day: carbohydrates, 60%-67.5%; fat, 21%-24.2%; protein, 11.2%-18.4%) and insulin glargine with metformin. The metformin dosage was set as high as possible after considering the patient’s age, renal function, and other adverse effects. Metformin was not administered to subjects who were older than 75 years of age or who had a serum creatinine level higher than 1.3 mg/dL (for men) or 1.2 mg/dL (for women).

For the basal insulin therapy, insulin glargine was used. The dose was titrated at the physician’s discretion to achieve a target fasting blood glucose (FBG) level between 70–130 mg/dL. An evaluation of the daily glucose profiles indicated that at least 5 days after admission were required to regulate the FBG at the target level. If the postprandial 2-hour blood glucose (PBG) level corresponded to the target value (lower than 180 mg/dL), the study protocol ended (STEP 0 responder). Patients who did not achieve the target PBG value were additionally treated with postprandial hypoglycemic agents (refer to STEP 1 and STEP 2 below). The target PBG was defined according to the American Diabetes Association’s recommendation [16].

#### ***STEP 1***

On day 1, miglitol (150 mg/day, 50 mg administered immediately before every meal) was administered and the PBG was evaluated. If the target PBG was not achieved on day 1, the dose of miglitol was increased to 225 mg/day (75 mg administered immediately before every meal) on day 2. If the patient experienced gastrointestinal symptoms, the dose of miglitol was decreased to a tolerable dose on day 3. The PBG was evaluated on day 2 or day 3. If the PBG was under 180 mg/dL, the study protocol was regarded as having been successful at STEP 1.

#### ***STEP 2***

For patients who failed to achieve the target PBG with miglitol, mitiglinide (30 mg/day, 10 mg administered immediately before every meal) was added to the treatment regimen on day 4 (or day 5). If the target PBG was not reached, the dosage of mitiglinide was increased to 60 mg/day (20 mg administered immediately before every meal) on day 5 (or day 6) and the PBG was evaluated.

### Biochemical and clinical measurements

We calculated the BMI using the body height and weight of each patient. The 24-h urine C peptide and serum C peptide levels were measured before and 6 minutes after the intravenous injection of 1 mg of glucagon (glucagon stimulation test).

The self-monitoring blood glucose (SMBG) levels were checked before and 2 hours after each meal and before sleep (7-point SMBG) using a Nipro StatStrip XP (Nova Biomedical K.K.). The M-value was calculated using the following equation:

$$\begin{array}{l} M‒\text{value}=M_{\mathrm{GR}}+M_{W},\\ \;M_{\mathrm{GR}}=\frac{{\overset{t_{k}}{\underset{t=t_{1}}{\sum }}\left|{}^{10}\text{log}\frac{{\mathrm{BS}}_{t_{i}}}{100}\right|}^{3}}{7},M_{W}=\frac{G_{\text{max}}‒G_{\text{min}}}{20} \end{array}$$

(M_GR_ = M-value for glucose reading, M_W_ = correction factor, BS_ti_ = glucose reading at time t_i_, G_max_ = maximum glucose reading, G_min_ = minimum glucose reading) [17,18].

The patients were observed using continuous glucose monitoring (CGMS® System Gold™; Medtronic Inc.); the CGMS device was calibrated four times a day. The results were then used to calculate the 24-h average, the standard deviation (SD) of the 24-h values, the 24-h area under the curve (AUC), every 4-h postprandial value, and from midnight to early morning (from 0:00 AM to 8:00 AM), and the mean amplitude of glycemic excursion (MAGE [19]) using the glucose values observed every 5 minutes. We failed to obtain CGM records in 2 of the 16 patients because of technical difficulties.

### Primary and secondary outcomes

The primary endpoint was the change in the proportion of patients who reached the target PBG before and after the addition of the postprandial hypoglycemic agents (STEP 0 vs. end of protocol). We also calculated the M-value, MAGE, and the averages and SDs of the CGM data obtained on the days on which each of the steps were evaluated. We also compared the patient backgrounds of those in whom the treatment was or was not a success.

### Statistical analysis

Exact McNemar test was used to compare the proportion of patients who achieved the target PBG at STEP 0 and at the end of the protocol. Continuous variables were compared using the Wilcoxon rank sum test. A two-sided *P* value <0.05 was regarded as significant. The results were described using the median and interquartile ranges. The statistical analysis was performed using Stata IC 11.

## Results

**Figure 1:**
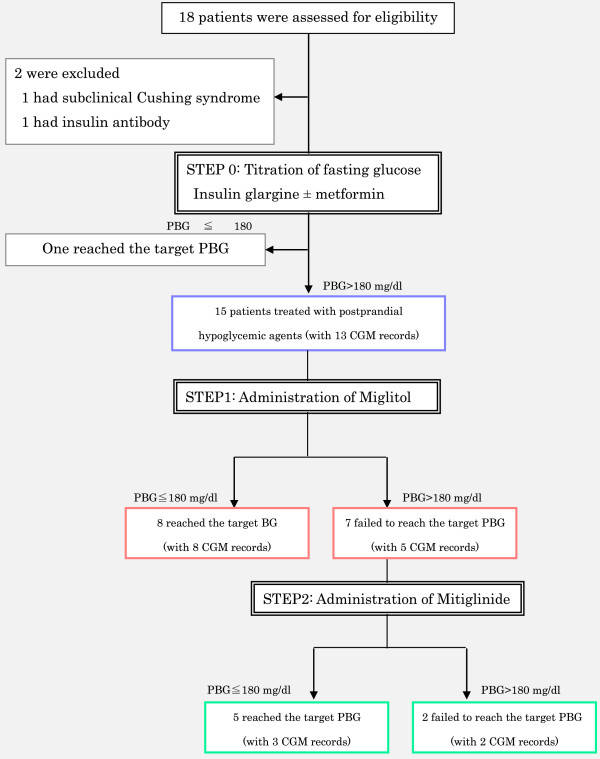
Enrollment and outcomes.

**Figure 2:**
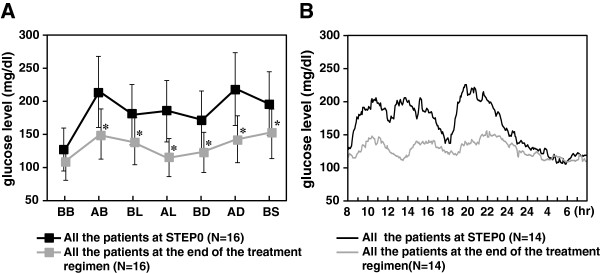
**Median 7**-**point SMBG and CGM of all the patients at STEP0 and at the end of the treatment regimen. A**. Median 7-point SMBG of all the patients at STEP 0 and at the end of the treatment regimen. Black line: all the patients at STEP 0, Gray line: all the patients at the end of the treatment regimen. BB: before breakfast, AB: after breakfast, BL: before lunch, AL: after lunch, BD: before dinner, AD: after dinner, BS: before sleep. *value of SMBG of all the patients at STEP 0 vs. those at the end of the treatment regimen *P* < 0.05. **B**. Median CGM of all the patients at STEP 0 and at the end of the treatment regimen. Black line: all the patients at STEP 0, Gray line: all the patients at the end of the treatment regimen.

We enrolled 18 patients with type 2 diabetes who had been hospitalized in our institution. Two patients were excluded: one patient was diagnosed as having preclinical Cushing syndrome, and the other was excluded because of the presence of an insulin autoimmune antibody. One patient reached the target PBG at STEP 0 and did not proceed to the next step in the protocol (Figure 1) (Table 1). Of the 15 patients who were treated with miglitol, 8 patients reached the target PBG at the STEP 1 evaluation. Mitiglinide was then added to the treatments of the 7 patients who failed to reach the target PBG using the STEP 1 treatment; 5 of these patients achieved the target PBG at the STEP 2 evaluation. Finally, 1 of the 16 patients achieved the target PBG at STEP 0 and 14 patients achieved the target PBG at the end of the study protocol (6.25% vs. 87.5%, *P* = 0.0002). The 7-point SMBG and CGM were also improved (Figure 2). The characteristics of the patients who reached the target PBG at STEP 1 and the patients who proceeded to STEP 2 are presented in Additional file 1: Table S1.

**Table 1:** **Characteristic of patients **(**N** = **16**)

***Demographics***	
Age (years)^a^	67.0 (58.0-71.0)
Sex (men/women)	11/5
BMI (kg/m^2^)^a^	25.0 (22.0-27.9)
Duration of diabetes (years)^a^	14.0 (8.5-24.75)
HbA1c (%)^a^	9.1 (8.3-10.4)
Glycated albumin (%)^a^	22.9 (19.8-26.8)
eGFR (mL/min/1.73 m^2^)^a^	70.0 (62.6-82.4)
Previous medication of diabetes	
Insulin^b^	7
Oral hypoglycemic agents	
Sulfonylurea^b^	5
Metformin^b^	9
Alpha-glucosidase inhibitor^b^	4
Glinide^b^	2
DPP-4 inhibitor^b^	0
Administration of metformin at STEP 0^b^	10

^a^: Median (interquartile range), ^b^: Numbers.

**Figure 3:**
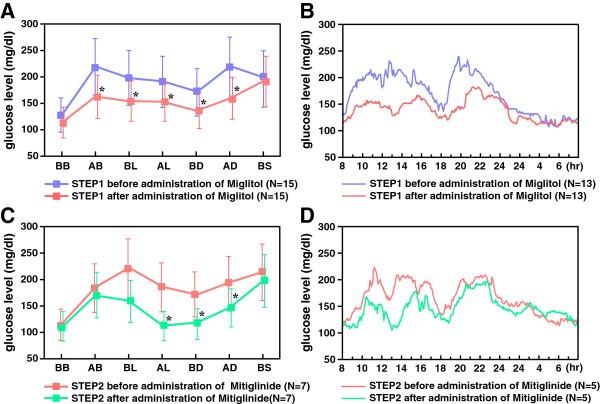
**Median 7**-**point SMBG and CGM at STEP 1 and STEP 2. A**. Median 7-point SMBG of patients who proceeded to STEP 1 (before and after administration of Miglitol). Blue line: The patients who proceeded to STEP 1 before administration of Miglitol, Red line: The patients who proceeded to STEP 1 after administration of Miglitol. BB: before breakfast, AB: after breakfast, BL: before lunch, AL: after lunch, BD: before dinner, before AD: after dinner, BS: before sleep. *value of SMBG of patients who proceeded to STEP 1 before vs. after administration of Miglitol *P* < 0.05. **B**. Median CGM of patients who proceeded to STEP1 (before and after administration of Miglitol). Blue line: The patients who proceeded to STEP1 before administration of Miglitol, Red line: The patients who proceeded to STEP1 after administration of Miglitol. **C**. Median 7-point SMBG of patients who proceeded to STEP 2 (before and after administration of Mitiglinide). Red line: The patients who proceeded to STEP 2 before administration of Mitiglinide, Green line: The patients who proceeded to STEP 2 after administration of Mitiglinide. BB: before breakfast, AB: after breakfast, BL: before lunch, AL: after lunch, BD: before dinner, AD: after dinner, BS: before sleep. *value of SMBG of patients who proceeded to STEP 2 before vs. after administration of Mitiglinide *P* < 0.05. **D**. Median CGM of patients who proceeded to STEP 2 (before and after administration of Mitiglinide). Red line: The patients who proceeded to STEP 2 before administration of Mitiglinide. Green line: The patients who proceeded to STEP 2 after administration of Mitiglinide.

At STEP 0, the 7-point glucose monitoring values exhibited sustained levels of post-breakfast hyperglycemia. With the administration of miglitol, the 7-point glucose monitoring values from after breakfast until after dinner were suppressed, but the values before sleep and before breakfast did not decrease significantly (Figure 3A). When the glycemic profiles were examined more precisely using CGM, the 24-h AUC and each of the 4-h postprandial AUC values were significantly decreased. The AUC from 0:00 AM to 8:00 AM (from evening until before breakfast) remained unchanged at the STEP1 evaluation after the addition of miglitol, compared with the values at STEP0 (Figure 3B).

Similarly, compared with STEP 1, the 7-point glucose monitoring values from after lunch through until after dinner were significantly decreased at the STEP 2 evaluation (Figure 3C), the same day of CGM profile was described (Figure 3D). The 24-h AUC, the 4-h after-lunch AUC, and the 4-h after dinner AUC recorded using CGM were significantly decreased at the STEP 2 evaluation. The details of the CGM data for each step are described in an Additional file 2: Table S2.

The markers of glucose fluctuations, namely the SDs of the glucose levels for 24-h and MAGE, improved and the M-values were also decreased at the end of protocol (Table 2).

**Table 2:** Results of area under the curve of CGM and glucose fluctuations at STEP 0 and at the end of this protocol

	***At the evaluation of STEP 0***	***At the end of this protocol***
**Results of continuous glucose monitoring**		
Number of patients	14	14
AUC for 24-h glycemic fluctuation (mg・h/dL)	4112.1	2986.3*
	(3568.8-4338.5)	(2843.3-3504.0)
AUC for 4-h after breakfast glycemic fluctuation (mg・h/dL)	732.3	521.5^*^
	(607.4-845.4)	(498.9-626.9)
AUC for 4-h after lunch glycemic fluctuation (mg・h/dL)	785.9	516.2^*^
	(573.5-952.0)	(466.5-550.6)
AUC for 4-h after dinner glycemic fluctuation (mg・h/dL)	785.3	559.2^*^
	(719.4-877.8)	(485.3-651.4)
AUC for 8-h from midnight to early morning glycemic fluctuation (00:00–08:00) (mg・h/dL)	1033.0	949.8
	(911.4-1187.5)	(869.6-1074.3)
24-h mean glucose levels (mg/dL)	169.7	124.7^*^
	(142.6-181.1)	(118.8-146.4)
SDs of 288 glucose levels for 24-h (mg/dL)	38.6	23.5*
	(29.9-58.5)	(13.9-28.4)
Proportion of time (%) in hyperglycemia (>180 mg/dL)	40.7	7.9^*^
	(17.4-53.7)	(0–17.2)
Proportion of time (%) in hypoglycemia (<70 mg/dL)	0	0
MAGE	91.1 (79.1-119.6)	61.3^*^ (50.2-72.4)
**Results of 7**-**point SMBG**		
Number of patients	16	16
M-value	28.8 (21.4-52.5)	8.7 ^*^(5.0-15.7)

Median (interquartile range)

*P < 0.05

CGM data were analyzed in a total of 14 patients because of missing CGM data in 2 of the 16 patients.

Two patients were unable to achieve the target PBG. One patient was a 78-year-old man; although he exhibited a comparably sufficient endogenous insulin level, he was obese and could not use metformin because of his age. The other patient was a 61-year-old woman; she had a long duration of diabetes, and her endogenous insulin level was almost depleted. Both of these patients had highly fluctuating glucose levels, as shown by the M-value and the SDs of the 24-h data and MAGE obtained using CGM (Table 3).

**Table 3:** Characteristics of patients who did or did not reach the target PBG

	***Patients who reached the target PBG using this protocol *** **(*N*** **=** ***14*)**	***Patients who failed to reach the target PBG***	
		***①***	②
Age (years)	67.0 (55.5-70.5)	78	61
Sex (men/women)	10/4	man	woman
BMI (kg/m^2^)	25.0 (21.8-27.8)	28.0	22.1
Duration of diabetes (years)	13.0 (7.0-21.5)	12	40
HbA1c (%)	9.2 (8.7-11.2)	8.3	8.2
Glycated albumin (%)	23.0 (20.2-29.4)	21.1	19.8
eGFR (mL/min/1.73 m^2^)	70.5 (58–84.2)	70.0	67.2
Urinary C-peptide excretion (μg/day)	43.5 (24.8-69.6)	67.4	14.4
C-peptide			
(Glucagon stimulated test)			
at 0 minutes (ng/mL)	0.9 (0.75-1.45)	1.9	0.4
at 6 minutes (ng/mL)	2.3 (1.45-2.9)	3.0	0.8
Δ (ng/mL)	1.1 (0.65-1.40)	1.1	0.4
Insulin glargine (units/day)	14 (8.5-18.5)	12	13
Insulin glargine (units/kg)	0.2 (0.17-0.26)	0.15	0.23
MAGE at the final CGM evaluation	60.5 (46.9-69.2)	61.3	87.6
	(N = 12)		
M-value at the final SMBG evaluation	8.4 (4.3-14.1)	24.2	18.0

Median (interquartile range).

No severe hypoglycemic events occurred. Regarding the CGM data, hypoglycemia of less than 70 mg/dL was rarely recorded from midnight until daytime (Table 2).

## Discussion

Postprandial hyperglycemia can have a harmful effect on the cardiovascular outcome. In this study, ten patients were treated with as high a dose of metformin as they could tolerate; metformin is recommended as a drug of first choice [16] in combination with insulin glargine at STEP 0. The remaining six patients were treated with insulin glargine alone at STEP 0 because of an advanced age or a reduced renal function. The administration of basal insulin with or without metformin decreases the fasting glucose level and the overall glycemic profile, but in our cases, it was insufficient to postprandial hyperglycemia in 15 of the 16 cases. Many studies have discussed the combination of basal insulin with metformin or sufonylurea or both drugs [5,20-22]. However, their mechanisms of action can make them insufficient to prevent postprandial hyperglycemia. Consequently, we prescribed miglitol and mitiglinide as a two-step treatment. Using our treatment regimen, 14 of the 16 patients achieved the target PBG as defined by the American Diabetes Association’s recommendations [16]. Though the duration of the hospital stay might have contributed to the gradual improvement in the glucose level and might have influenced the achievement of the target PBG, the fact that most of the subjects eventually attained an ideal glycemic control using this regimen is noteworthy. In addition, CGM highlighted the precise profiles of glucose excursion, showing the effectiveness of the miglitol and mitiglinide in lowering daytime blood glucose levels without causing hypoglycemia during the night as well as showing the improvement in glucose fluctuations as a result of treatment with the two drugs in more detail.

The two patients who were unable to attain the target PBG suggest that the present regimen has some limitations. One patient was an obese, elderly man who was unable to take metformin because of his age. In this patient, insulin resistance might have limited the effect of mitiglinide. The other patient was a woman with the lowest serum C-peptide at 6 minutes in the glucagon test. For this patient, the effect of mitiglinide might have been limited by the impaired secretion of insulin.

In this study, we used two postprandial hypoglycemic agents. Alpha-glucosidase inhibitors, which delay the absorption of carbohydrates in food, decrease the postprandial glucose level without inducing the secretion of insulin [23]. As a result of this unique mechanism, alpha-glucosidase is effective for patients even if they have a comparatively long duration of diabetes and a severe deterioration of insulin secretion. In addition, miglitol provides incretin effects induced by GLP-1 secretion from L-cells [24]. Glinide is a short-acting insulin secretagogue that functions through the K_ATP_ channel inhibition of β cells [25]. Mitiglinide also suppresses postprandial free fat acid levels [26]. These drugs decrease postprandial hyperglycemia effectively while causing fewer episodes of hypoglycemia and are relatively safe for patients with renal dysfunction and for elderly patients, in addition to being safe for long-term use.

Numerous studies have supported the effectiveness of basal insulin with sulfonylurea or metformin [5,20-22] but as far as we know, few studies have examined the use of insulin glargine with postprandial hypoglycemic agents. Kim et al. reported the efficacy of adding either nateglinide or acarbose to insulin glargine, resulting in a 7-point reduction in the SMBG and with no difference in glucose variability between nateglinide and acarbose [27]. Hirose et al. investigated the effect of mitiglinide with insulin glargine after switching from multiple daily injections and reported that 15 out of 30 patients maintained a fair glucose control after switching to mitiglinide [28,29]. These reports suggested the effectiveness of using postprandial hypoglycemic agents with long-acting basal insulin and also indicated the limitations of the addition of single postprandial hypoglycemic agents.

To improve postprandial hypoglycemia, dipeptidyl peptidase-4 (DPP-4) inhibitors and GLP-1 analogs are other choices for use in combination with basal insulin [30,31]. We are also attempting to construct new regimens composed of basal insulin and multiple hypoglycemic agents, including DPP-4 inhibitors or GLP-1 analogs [32].

Initiating basal insulin therapy in patients with poor glycemic control after the use of metformin is supported by the American Diabetes Association [33,34] and the European Association for the Study of Diabetes [34]. In addition, this treatment regimen may lead to better glycemic control, compared with only basal insulin with metformin, because both the fasting and postprandial blood glucose levels can be controlled. This regimen might also serve as a go-between for frequent insulin injections therapy.

This study had several limitations including the relatively small number of subjects, the absence of a control group, and the short-term results for inpatients. The patients’ satisfaction level after receiving this regimen and long-term diabetic complications, cardiovascular disease, and mortality also need to be evaluated. In addition, larger studies are needed to evaluate the reduction of hypoglycemia associated with this regimen. Confirming this hypothesis, further studies are required to confirm the efficacy of this regimen in outpatients; furthermore, the regimen should be simplified before being administered to outpatients.

Once such points have been clarified, our structured treatment regimen might be beneficial for many type 2 diabetes patients requiring insulin therapy.

## Conclusions

In conclusion, in a pilot study of a two-step regimen consisting of the addition of postprandial hypoglycemic agents, more than 80% of the patients achieved a good glucose profile, indicating the potent efficacy of the two-step administration of postprandial hypoglycemic agents with basal insulin therapy.

## Abbreviations

OHAs: Oral hypoglycemic agents; CGM: Continuous glucose monitoring; SMBG: Self-monitoring blood glucose; FBG: Fasting blood glucose; PBG: Postprandial 2-hour blood glucose; SDs: Standard deviations; AUC: Area under the curves; DPP-4 inhibitor: Dipeptidyl peptidase-4 inhibitor; eGFR: Estimated glomerular filtration rate.

## Competing interests

The authors declare that they have no competing interests.

## Authors’ contributions

NI conceptualized the idea for the study, collected the data, performed the statistical analysis, and wrote the paper. TT participated in the design of the study, joined the discussion, and was involved in drafting the manuscript. R Y-H participated in the design of the study, joined the discussion, and was involved in drafting the manuscript. MKishimoto jointed the discussion and was involved in drafting the manuscript. HK and HN joined the discussion. MKakei joined the discussion and supervised the work. MN participated in the design of the study, supervised the work, and joined the discussion. All authors have read and approved the final manuscript.

## Supplementary Material

Additional file 1: Table S1 Characteristics of the patients who reached the target PBG at STEP 1 and the patients who proceeded to STEP2. Click here for file

Additional file 2: Table S2 Detailed results of area under the curve for Continuous Glucose Monitoring and glucose fluctuation STEP 1 and STEP 2. Click here for file
