# Supplementary material for: Improvement of both fasting and postprandial glycemic control by the two-step addition of miglitol and mitiglinide to basal insulin therapy: a pilot study
Source: Diabetol Metab Syndr. 2014 Mar 31;6:48. doi: 10.1186/1758-5996-6-48 (PMC4025538; doi:10.1186/1758-5996-6-48)
Supplement: Additional file 1: Table S1 — Characteristics of the patients who reached the target PBG at STEP 1 and the patients who proceeded to STEP2. [file 1758-5996-6-48-S1.doc]

**Additional file 1:** Table S1. Characteristics of the patients who reached the target PBG at STEP 1 and the patients who proceeded to STEP2.

|  | *Patients who reached the target PBG at STEP1*  *(N=8)* | *Patients who didn’t reach the target PBG at STEP1 and proceeded to STEP 2 (N=7)* |
| --- | --- | --- |
| Age (years) | 59.5(56.7-70.2) | 70.0(67.5-74.5) |
| Sex (men/women) | 6/2 | 4/3 |
| BMI (kg/m^2^) | 24.4(21.9-27.6) | 22.5(21.7-28.0) |
| Duration of diabetes (years) | 10.0(4.0-16.2) | 24.5(14.5-37.5) |
| HbA1c (%) | 11.2(9.7-12.7) | 8.9(8.2-9.1) |
| Glycated albumin (%) | 28.1(22.5-34.3) | 22(20.1-22.9) |
| eGFR (mL/min/1.73 m^2^) | 74.7(63.4-91.2) | 67.2(58.7-69.0) |
| Urinary C-peptide excretion  (µg/day) | 60.2(39.3-67.6) | 21.9(18.1-53.7) |
| Glucagon stimulated test  C-peptide  at 0 minutes (ng/mL)  at 6 minutes (ng/mL)  Δ(ng/mL) | 1.1(0.90-1.77)  2.1(1.50-2.77)  0.85(0.57-1.22) | 0.6(0.50-1.55)  1.7(1.05-2.80)  1.1(0.55-1.25) |
| Insulin glargine (units/day)  (units/kg) | 14.5(8.7-16.5)  0.21(0.18-0.23) | 13(9-17)  0.23(0.13-0.29) |

Median (interquartile range)
