# Supplementary material for: Improvement of both fasting and postprandial glycemic control by the two-step addition of miglitol and mitiglinide to basal insulin therapy: a pilot study
Source: Diabetol Metab Syndr. 2014 Mar 31;6:48. doi: 10.1186/1758-5996-6-48 (PMC4025538; doi:10.1186/1758-5996-6-48)
Supplement: Additional file 2: Table S2 — Detailed results of area under the curve for Continuous Glucose Monitoring and glucose fluctuation STEP 1 and STEP 2. [file 1758-5996-6-48-S2.doc]

Additional file 2: Table S2. Detailed results of area under the curve for Continuous Glucose Monitoring and glucose fluctuation STEP 1 and STEP 2

|  | ***At the evaluation of STEP 1 before the administration of Miglitol*** | ***At the evaluation of STEP 1 after the administration of Miglitol*** | ***At the evaluation of STEP 2 before the administration of Mitiglinide*** | ***At the evaluation of STEP 2 after the administration of Mitiglinide*** |
| --- | --- | --- | --- | --- |
| **Results of continuous glucose monitoring** |  |  |  |  |
| Number of patients | 13 | 13 | 5 | 5 |
| AUC for 24-h glycemic fluctuation (mg・h/dL) | 4295.5 (3813.2-4342.7) | 3531.3^a^ (2868.7-3992.9) | 4075.6 (3702.9-4583.8) | 3422.0^b^ (3207.1-3759.7) |
| AUC for 4 h after breakfast glycemic fluctuation (mg・h/dL) | 740.0 (612.8-853.8) | 573.7^a^ (520.8-721.0) | 612.4 (573.7-762.9) | 510.2 (502.0-608.5) |
| AUC for 4 h after lunch glycemic fluctuation (mg・h/dL) | 792.0 (627.1-967.5) | 554.0^a^ (499.9-752.1) | 822.3 (730.9-839.2) | 540.5^b^ (513.2-604.9) |
| AUC for 4 h after dinner glycemic fluctuation (mg・h/dL) | 794.7 (738.6-887.2) | 638.5^a^ (485.5-682.3) | 682.3 (674.0-816.6) | 655.7^b^ (616.1-679.0) |
| AUC for 8 h from midnight to early morning glycemic fluctuation (00:00-08:00 ) (mg・h/dL) | 1054.0 (917.6-1201.9) | 974.9 (867.1-1076.3) | 1095.0 (1014.4-1409.8) | 1130.9 (1041-1161.8) |
| 24-h mean glucose levels (mg/dL) | 179.3 (145.7-181.3) | 147.6^a^ (119.9-167.7) | 170.2 (154.6-191.4) | 142.9^b^ (133.9-157.1) |
| SDs of 288 glucose levels for 24 h (mg/dL) | 42.9(30.6-59.5) | 27.8 ^a^ (20.5-37.5) | 40.9(35.7-47.3) | 31.3(21.7-34.2) |
| Proportion of time (%) in hyperglycemia (>180 mg/dL) | 43.75 (22.9-54.8) | 11.8^a^ (0-39.5) | 39.5 (24.6-57.9) | 19.0 (11.1-19.7) |
| Proportion of time (%) in hypoglycemia (<70 mg/dL) | 0 | 0 | 0 (0-0.34) | 0 |
| MAGE | 100.2 (78.3-121.0) | 61.3^a^ (52.0-80.66) | 86.3 (66.1-101) | 67.6 (61.3-87.6) |
| **Results of 7-point SMBG** |  |  |  |  |
| Number of patients | 15 | 15 | 7 | 7 |
| M-value | 31.7 (23.3-53.1) (N = 15) | 15.7 ^a^ (8.2-28.9)(N = 15) | 32.1 (24.3-38.2)(N = 7) | 14.7 ^b^ (8.4-20.0)(N = 7) |

Median (interquartile range)

^a^ before administration of Miglitol vs. after administration of Miglitol at STEP 1, *P* < 0.05, ^b^ before administration of Mitiglinide vs. after administration of Mitiglinide at STEP 2, *P* < 0.05

CGM data were analyzed in a total of 14 patients because of missing CGM data in 2 of the 16 patients.
